# Surgical Education for Pressure Injuries: A Survey of What Residents are Learning in Ontario

**DOI:** 10.1177/22925503251410231

**Published:** 2026-01-06

**Authors:** Hoyee Wan, Romain Laurent, Alan Rogers, David Wallace

**Affiliations:** 1 Ross Tilley Burn Centre, 483372 Sunnybrook Health Sciences Centre, Toronto, Canada; 2 Division of Plastic, Reconstructive and Aesthetic Surgery, Department of Surgery, University of Toronto, Toronto, Canada

**Keywords:** pressure injury, surgical education, residency training, debridement, reconstruction, escarres, formation chirurgicale, résidence, débridement, reconstruction

## Abstract

**Background:** The optimal surgical management of complex pressure injuries (PIs) relies on adequate education during residency. However, both classroom-based and clinical exposure to these injuries may be inconsistent across training programs. This study aimed to evaluate Ontario surgical residents’ exposure to PI management and identify deficiencies in current curricula. **Methods:** A cross-sectional survey was developed and distributed to general, orthopaedic, and plastic surgery residents across Ontario. The survey collected data on didactic and clinical exposure to PI management, confidence in debridement and reconstruction, and perceptions of current training. Statistical analyses included descriptive statistics and comparisons between surgical specialties. **Results:** Forty-nine responses were obtained (response rate = 14%). Results demonstrated limited didactic and clinical exposure to PI management in nonplastic surgery residency programs. Most nonplastic surgery residents did not anticipate feeling comfortable performing PI debridement as part of their future practice following residency completion. In contrast, plastic surgery residents reported greater confidence in performing debridement but lacked confidence in selecting appropriate surgical candidates for reconstruction. **Conclusions:** Exposure to PI reconstruction for plastic surgery residents appears to be limited. Both nonplastic and plastic surgery residents expressed strong interest in additional educational initiatives focused on the surgical management of PIs. This study highlights the need for enhanced educational opportunities, including structured curricula and increased clinical exposure, to ensure surgical trainees develop the necessary competencies for managing PIs effectively.

## Résumé

**Introduction :** La prise en charge chirurgicale optimale des plaies de pression (PP) complexes repose sur une formation adéquate durant la résidence. Toutefois, l’exposition théorique et clinique à ces lésions reste inégale selon les programmes. Cette étude visait à évaluer l’exposition des résidents en chirurgie de l’Ontario à la gestion des PP et à identifier les lacunes des programmes actuels. **Méthodes :** Un sondage transversal a été élaboré et diffusé auprès des résidents en chirurgie générale, orthopédique et plastique en Ontario. Il recueillait des données sur l’enseignement théorique et clinique relatif aux PP, la confiance des résidents quant au débridement et à la reconstruction, ainsi que leur perception de la formation reçue. Les données ont été analysées par statistiques descriptives et comparatives entre spécialités. **Résultats :** Quarante-neuf réponses ont été obtenues (taux de réponse : 14%). L’étude révèle une exposition limitée à la gestion des PP dans les programmes hors chirurgie plastique. La majorité des résidents non plasticiens ne s’estimaient pas à l’aise de réaliser un débridement dans leur future pratique. En revanche, les résidents en chirurgie plastique affichaient une plus grande aisance pour le débridement, mais un manque de confiance dans la sélection des candidats à la reconstruction. **Conclusions :** L’exposition à la reconstruction des PP semble, même en chirurgie plastique. Tous les groupes ont exprimé un fort intérêt pour des initiatives éducatives ciblées. Cette étude souligne la nécessité d’un curriculum structuré et d’une exposition clinique plus importante pour garantir le développement des compétences requises à la gestion des PP.

## Introduction

Pressure injuries (PIs), sometimes referred to as “pressure ulcers” or “decubitus ulcers,” represent a significant burden in Ontario hospitals and long-term care facilities, leading to increased patient morbidity, prolonged hospitalizations, and substantial healthcare costs.^1–4^ While prevention remains the primary strategy, many PIs progress to stages requiring surgical intervention.

Plastic surgeons are primarily responsible for the surgical management of complex PIs in Ontario. However, in hospitals with limited plastic surgery availability, general and orthopaedic surgeons may also play a role in managing these patients.^5,6^ Given the widespread prevalence of PIs, it is imperative that residents across multiple surgical disciplines receive adequate training in PI diagnosis, staging, and surgical intervention.

Although the medical literature describes the surgical management of PIs,^7^ variability in residency training influences the level of exposure and competency among trainees. The goal of this study is to assess Ontario surgical residents’ educational exposure to PI management and identify gaps that may influence their ability to provide effective surgical care.

## Methods

This cross-sectional study surveyed general, orthopaedic, and plastic surgery residents in Ontario regarding their exposure to PI management. A web-based survey was developed using SurveyMonkey (Momentive, San Mateo, California) and was approved by the Redacted for anonymity Research Ethics Committee (REB-5990) (see Supplemental Questionnaire). The survey was distributed to all 7 Ontario residency-training programs and included 20 multiple-choice and free-text questions eliciting demographic information, PI identification, staging, surgical management, postoperative care, and self-assessed competency.

Participation was voluntary and anonymous. Informed consent was obtained prior to study participants completing the survey. To encourage engagement, 3 $100 gift cards were offered as incentives. The survey was initially distributed on March 26, 2024, via program administrators, with 2 follow-up emails over the subsequent month. The final submission deadline was April 30, 2024. Descriptive statistics were used to summarize the data, with categorical variables presented as frequencies and percentages.

## Results

Forty-nine residents of 351 participated (response rate, 14%), representing all surgical specialties except for plastic surgery and orthopaedic surgery at 1 institution. Respondents included 16 general surgery, 19 orthopaedic surgery, and 14 plastic surgery residents, spanning all training levels (post-graduate year [PGY]1-5) (Supplemental Table 1).

**Figure 1.**
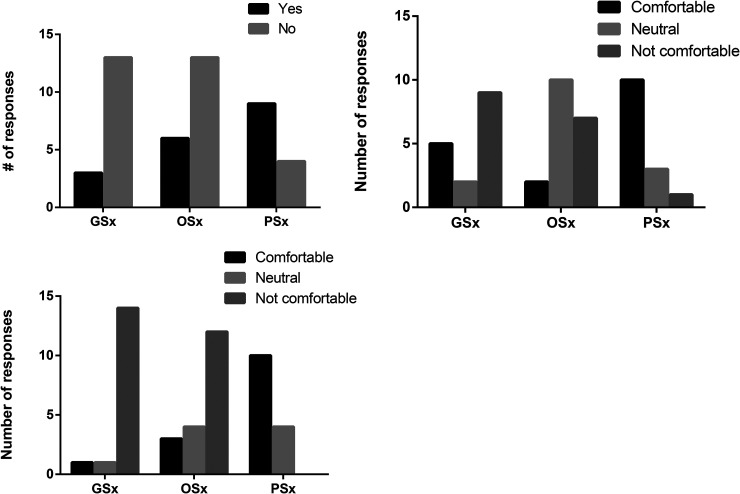
Resident experiences with didactic teaching and comfort level surrounding diagnosing PI and identifying candidates for PI surgery. (A) Have you received any didactic teaching related to pressure injuries? (B) Do you feel comfortable diagnosing and staging common pressure injuries (eg, sacral, ischial, trochanteric)? (C) Do you feel comfortable identifying appropriate candidates for pressure injury surgery (debridement or reconstruction)? Abbreviations: PI, pressure injury; GSx, general surgery; OSx, orhopaedic surgery; pSx, plastic surgery).

Most general (13 of 16; 81%) and orthopaedic (13 of 19; 68%) surgery residents reported receiving no formal didactic teaching on PI management (Figure 1A). Among those who had received instruction, exposure was minimal (<1 h). In contrast, a majority of plastic surgery residents (9 of 14; 64%) had received at least 2 h of instruction.

Regarding diagnostic competence, general surgery residents largely reported discomfort with PI staging (Figure 1B). Orthopaedic residents were generally neutral or uncomfortable, while plastic surgery residents were mostly confident (10 of 14; 71%). Identifying surgical candidates for PI procedures posed a greater challenge, with nearly all nonplastic surgery residents expressing discomfort (Figure 1C). Finally, in terms of medical management of patients with pressure ulcers, all plastic surgery residents were comfortable (14 of 14; 100%), while the majority of orthopaedic residents and general surgery residents were uncomfortable (orthopaedic: 7 of 19; 38%, general surgery: 10 of 16; 62.5%).

**Figure 2.**
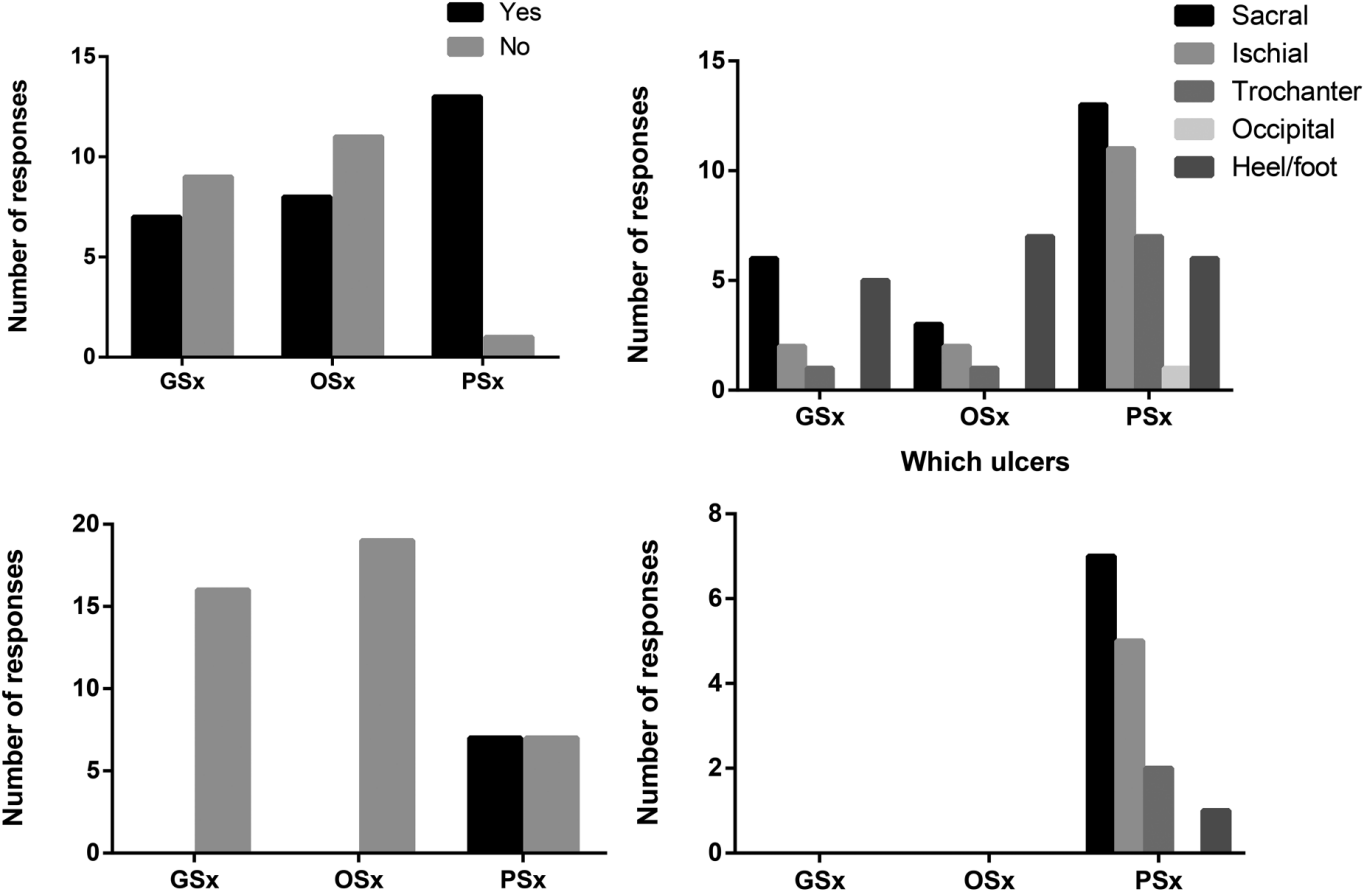
Resident experiences with debriding and reconstructing pressure injuries. (A) During your residency training, have you debrided any pressure injuries? (B) Which injuries have you debrided? (C) During your residency training, have you reconstructed any pressure injuries? (D) Which parts of the body have you reconstructed pressure injuries?

**Figure 3.**
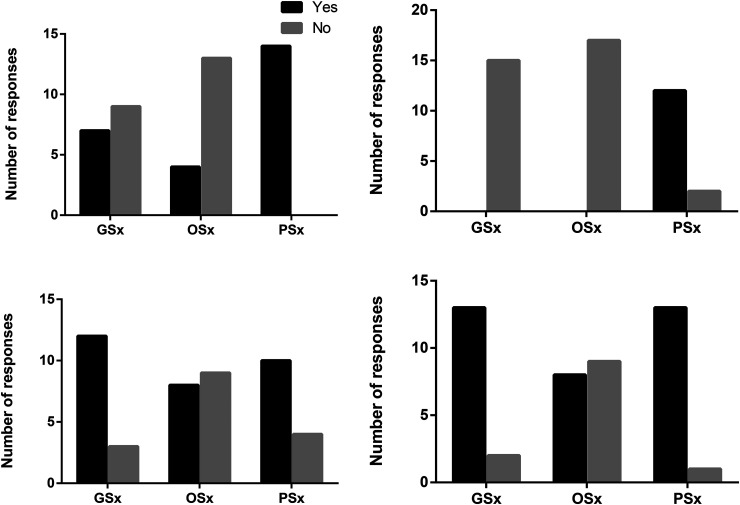
Expected level of competence after residency on debriding and reconstruction pressure injuries and interest in education around PI. (A) By the end of residency, do you think you will be comfortable debriding pressure injuries as part of your practice? (B) By the end of residency, do you think you will be comfortable reconstructing pressure injuries as part of your practice? (C) Would you be interested in more exposure to pressure injury education during your training program? (D) Would you see value in having a dedicated wound care and surgery elective? Abbreviation: PI, pressure injury.

Most plastic surgery residents (13 of 14; 93%) had experience with PI debridement, compared to (7 of 16; 44%) general and (8 of 19; 42%) orthopaedic surgery residents (Figure 2A). General surgery residents most frequently debrided sacral and heel/foot PIs, while orthopaedic residents primarily debrided heel/foot wounds (Figure 2B). All plastic surgery residents felt that they would be competent at debriding PIs after residency whereas as most orthopaedic (15 of 19; 79%) and general surgery residents (9 of 16; 56%) did not (Figure 3A).

Regarding reconstruction, nonplastic surgery residents had no exposure (Figure 2C). Among plastic surgery residents, only half (7 of 14; 50%) had performed PI reconstruction, predominantly for sacral and ischial wounds (Figure 2D). Most had reconstructed fewer than 5 cases. Despite this, 12 of 14 (85%) plastic surgery residents felt comfortable performing reconstruction postresidency (Figure 3B).

General (1 of 16; 6%) and orthopaedic (1 of 19; 5%) residents felt uncomfortable with postoperative rehabilitation planning, whereas most plastic surgery residents (12 of 14; 86%) were confident in this aspect.

Interest in additional PI education was strong across all groups: 12 of 16 (75%) general, 8 of 19 (42%) orthopaedic, and 10 of 14 (71%) plastic surgery residents expressed interest (Figure 3C). A majority also supported a dedicated wound care elective (Figure 3D).

## Discussion

Surgeons are responsible for managing complex PIs, yet the current educational framework for surgical trainees is insufficient to ensure competency in this field. The purpose of this study was to assess the state of PI education among surgical residents in Ontario. Our findings reveal a substantial disparity in exposure, confidence, and technical skills between plastic surgery and nonplastic surgery trainees. Notably, nonplastic surgery residents receive significantly less education on PIs, lack confidence in staging and identifying surgical candidates, and are less involved in acute surgical management. This highlights an opportunity to develop a structured PI curriculum tailored to the needs of surgical trainees.

Nonplastic surgery specialties frequently serve as the primary admitting service for patients with PIs, necessitating a foundational understanding of staging and identifying candidates for reconstruction. However, our study indicates that nonplastic surgery residents receive limited formal teaching on PIs and feel significantly less comfortable with staging and surgical decision-making than their plastic surgery counterparts. These findings are consistent with international data. A study in Australia found that only 40% of medical residents felt comfortable diagnosing and treating PIs,^8^ while research from India revealed that both general and plastic surgery residents reported inadequate knowledge in at least 70% of cases.^9^ To our knowledge, this is the first study in Canada assessing surgical trainee education on PIs. While orthopaedic and general surgery trainees report lower exposure and confidence, plastic surgery residents fare better—though gaps remain, particularly in identifying surgical candidates. This underscores the need for formal education programs that standardize PI training across surgical specialties.

The surgical management of soft tissue wounds falls within the scope of multiple specialties, yet our results demonstrate that nonplastic surgery trainees are neither trained nor comfortable in performing debridement. They report deficiencies in identifying surgical candidates and lack the technical skills to perform this fundamental surgical skill, which aligns with their limited formal education on PIs. This is concerning, as many Ontario hospitals require general or orthopaedic surgeons to perform initial debridement, with plastic surgery support available only when necessary. Adequate debridement is a fundamental surgical competency, as outlined in the Entrustable Professional Activities (EPAs) of general, orthopaedic, and plastic surgery.^10–12^ In Ontario, the first 2 years of residency represent the Surgical Foundations program, where core competencies like wound care and debridement are introduced. Although our sample included residents at different stages of training, responses from PGY1-2 residents help identify early gaps and support the improvement of their curriculum. The discrepancy in training suggests that a structured surgical wound rotation or selective clinical experience could improve surgical competency in PI management across all relevant specialties.

As expected, nonplastic surgery trainees had no exposure to PI reconstruction. However, even among plastic surgery residents, surgical experience was limited—only about half had participated in reconstructive procedures, primarily for sacral and ischial wounds. Notably, the reported case volumes were low, with most residents performing fewer than 5 reconstructive PI cases throughout residency. Despite this, many stated that they would be comfortable with these procedures upon graduation, raising concerns about whether current training provides adequate exposure. Given that plastic surgery is the specialty primarily responsible for PI reconstruction, it is imperative that training programs ensure appropriate case volume and mentorship. This aligns with Royal College of Physicians and Surgeons of Canada Plastic Surgery EPA #11, which mandates competency in managing complex wounds of the abdomen, trunk, and pelvis.^10^ Furthermore, practicing plastic surgeons in Canada have emphasized the need for targeted PI training to ensure competency in real-world practice.^13^ Given that most survey respondents expressed interest in a dedicated training program, formal mentorship with high-volume PI surgeons would be a logical next step to enhance surgical competency in this area.

Our study has several limitations. Firstly, while we received responses from across all programs, our low response rate introduces the possibility of nonresponse bias. Additionally, temporal and spatial biases exist—our survey was distributed toward the end of the academic year, meaning responses reflect a wide range of training levels. While it is expected that both didactic and practical experience increases over time, our sample size was insufficient to conduct meaningful comparisons between different levels of training. Furthermore, institutional resource disparities may affect PI education; for example, institutions without plastic surgery programs, such as Queen's University and the Northern Ontario School of Medicine, likely place greater responsibility for PI management on other surgical services. Finally, we attempted to capture data from residents across the province to describe differences between academic and community settings. However, the response rate precluded this, and any such observations remain subjective and unsupported.

## Conclusion

This study highlights gaps in PI education among surgical trainees in Ontario. A structured curriculum, increased clinical exposure, and interdisciplinary collaboration are necessary to ensure competency in PI diagnosis, debridement, and reconstruction. Efforts are underway to develop a dedicated wound care program to enhance surgical training in this critical area, ultimately improving patient outcomes and reducing healthcare costs.

## Supplemental Material

sj-docx-1-psg-10.1177_22925503251410231 - Supplemental material for Surgical Education for Pressure Injuries: A Survey of What Residents are Learning in Ontario Supplemental material, sj-docx-1-psg-10.1177_22925503251410231 for Surgical Education for Pressure Injuries: A Survey of What Residents are Learning in Ontario by Hoyee Wan, Romain Laurent, Alan Rogers and David Wallace in Plastic Surgery

sj-docx-2-psg-10.1177_22925503251410231 - Supplemental material for Surgical Education for Pressure Injuries: A Survey of What Residents are Learning in Ontario Supplemental material, sj-docx-2-psg-10.1177_22925503251410231 for Surgical Education for Pressure Injuries: A Survey of What Residents are Learning in Ontario by Hoyee Wan, Romain Laurent, Alan Rogers and David Wallace in Plastic Surgery
