# Supplementary material for: Surgical Education for Pressure Injuries: A Survey of What Residents are Learning in Ontario
Source: Plast Surg (Oakv). 2026 Jan 6:22925503251410231. Online ahead of print. doi: 10.1177/22925503251410231 (PMC12774815; doi:10.1177/22925503251410231)
Supplement: sj-docx-1-psg-10.1177_22925503251410231 - Supplemental material for Surgical Education for Pressure Injuries: A Survey of What Residents are Learning in Ontario [file sj-docx-1-psg-10.1177_22925503251410231.docx]

**Supplement**

**Questionnaire**

- 1. What training program are you in?
     1. Orthopedic Surgery, Plastic Surgery, General Surgery
  2. Program
     1. NOSM, University of Ottawa, Queen’s University, University of Toronto, McMaster University, Western University
  3. Year of training
     1. PGY1, PGY2, PGY3, PGY4, PGY5, PGY6+, Other – can put specific year as written text
  4. Identifying and staging
     1. Have you received any didactic teaching related to pressure injuries?
        1. Yes, No
     2. If yes, how many dedicated hours have you received during residency?
        1. 1h, 2h, >2h
     3. Do you feel comfortable diagnosing and staging common pressure injuries (e.g. Sacral, ischial, trochanteric)?
        1. Very uncomfortable, Uncomfortable, Neutral, Comfortable, Very comfortable
  5. Medical management
     1. Do you feel comfortable with medical (i.e., non-surgical) optimization of patients with pressure injuries to improve the surgical outcomes?
        1. Very uncomfortable, Uncomfortable, Neutral, Comfortable, Very comfortable
     2. Do you feel comfortable identifying appropriate candidates for pressure injury surgery (debridement or reconstruction)?
        1. Very uncomfortable, Uncomfortable, Neutral, Comfortable, Very comfortable
  6. Debridement
     1. During your residency training, have you debrided any pressure injuries?
        1. Yes, No
     2. If yes, which ones (select all that apply)?
        1. Sacral, Ischial, Trochanter, Occipital, Heel/Foot, Other
     3. If yes, wow many pressure injuries have you debrided?
        1. 1-5, 6-10, >10
  7. Reconstruction
     1. During your residency training, have you reconstructed any pressure injuries?
        1. Yes, No,
        2. If yes, which areas of the body(select all that apply)?
           1. Sacral, Ischial, Trochanter, Occipital, Heel/Foot, other
        3. If yes, how many pressure injuries have you reconstructed?
           1. 1-5, 6-10, >10
  8. Postoperative care
     1. Do you feel comfortable with principles of post-operative rehabilitation plans for pressure injury reconstruction?
        1. Yes, No
  9. Competency
     1. By the end of residency, do you think you will be comfortable debriding pressure injuries as part of your practice?
        1. Yes, No
     2. By the end of residency, do you think you will be comfortable reconstructing pressure injuries as part of your practice?
        1. Yes, No, Not applicable
  10. Future
      1. Would you be interested in more exposure to pressure injury education during your training program?
      2. Would you be interested in a dedicated wound care and surgery elective?
