# Supplementary material for: Surgical Education for Pressure Injuries: A Survey of What Residents are Learning in Ontario
Source: Plast Surg (Oakv). 2026 Jan 6:22925503251410231. Online ahead of print. doi: 10.1177/22925503251410231 (PMC12774815; doi:10.1177/22925503251410231)
Supplement: sj-docx-2-psg-10.1177_22925503251410231 - Supplemental material for Surgical Education for Pressure Injuries: A Survey of What Residents are Learning in Ontario [file sj-docx-2-psg-10.1177_22925503251410231.docx]

**Supplementary table I**

| **Specialty** | **School of origin** |  | **PGY year** |  |
| --- | --- | --- | --- | --- |
| **General Surgery** | McMaster | 4 | PGY1 | 7 |
|  | Northern Ontario School of Medicine | 3 | PGY2 | 5 |
|  | Queen’s University | 1 | PGY3 | 2 |
|  | University of Ottawa | 3 | PGY4 | 1 |
|  | University of Toronto | 2 | PGY5 | 1 |
|  | University of Western Ontario | 3 |  |  |
| **Orthopaedic Surgery** | McMaster | 9 | PGY1 | 4 |
|  | Northern Ontario School of Medicine | 0 | PGY2 | 5 |
|  | Queen’s University | 3 | PGY3 | 4 |
|  | University of Ottawa | 1 | PGY4 | 4 |
|  | University of Toronto | 5 | PGY5 | 2 |
|  | University of Western Ontario | 1 |  |  |
| Plastic Surgery | McMaster | 5 | PGY1 | 4 |
|  | Northern Ontario School of Medicine | No program | PGY2 | 1 |
|  | Queen’s University | No program | PGY3 | 2 |
|  | University of Ottawa | 1 | PGY4 | 4 |
|  | University of Toronto | 8 | PGY5 | 3 |
|  | University of Western Ontario | 0 |  |  |
